# Risk Factors for Early Hydrocephalus on Post Unilateral Thalamic Tumor Resection

**DOI:** 10.3389/fsurg.2022.814308

**Published:** 2022-04-08

**Authors:** Linpeng Zhang, Chen Wang, Xianwei Zeng

**Affiliations:** ^1^Department of Neurosurgery, Qilu Hospital, Cheeloo College of Medicine, Shandong University, Jinan, China; ^2^Department of Neurosurgery, Sanbo Brain Hospital, Capital Medical University, Beijing, China

**Keywords:** thalamic tumors, hydrocephalus, therapeutic method, surgery, OS, PFS

## Abstract

**Objective:**

The outcome of surgical treatment for thalamic tumors is poor. Hydrocephalus is one of the most frequent postoperative complications after unilateral thalamic tumor resection. This study examined the relationship between surgical approaches, pathological grade, image characteristics, preoperative complications, extent of resection, and incidence of postoperative hydrocephalus.

**Methods:**

The study retrospectively reviewed clinical data from 80 patients who underwent resection of thalamic tumors between 2015 and 2021. Data on patient survival and disease progression status were obtained retrospectively to calculate overall survival (OS) and progression free survival (PFS).

**Results:**

No patients died during the perioperative period and two patients suffered postoperative coma. Tumors were totally resected in 44 cases (55 %), subtotally resected in 21 cases (26.25 %), and partially resected in 15 cases (18.75 %). Thirty-five cases of hydrocephalus occurred within 1 month after operation(43.75%). Surgical approaches associated with hydrocephalus were as follows: hydrocephalus occurred in seven cases after trans-frontal lateral ventricle approach for tumor resection (62.9%), in 17 cases after through parieto-occipital transventricular approach tumor resection (43.58%), and in one case after trans-frontal lateral ventricle approach for tumor resection + third ventriculostomy (7.1%). Postoperative muscle strength decrease occurred in 41 patients (51.25%). Longer PFS and OS were correlated with degree of resection in patients with thalamic glioblastoma (*P* < 0.05) and had no relationship with hydrocephalus.

**Conclusion:**

Surgical treatment of thalamic tumors is an effective therapeutic method. The incidence of postoperative hydrocephalus is not associated with tumor size, degree of tumor enhancement, peritumoral edema, tumor invasion, midline crossing, and pathological grade. The incidence of postoperative hydrocephalus was higher in patients with preoperative hydrocephalus and low resection degree, and lower in patients with endoscopic third ventriculostomy. The risk of early postoperative hydrocephalus in thalamic tumors is high. Intraoperative third ventriculostomy could reduce the incidence of early postoperative hydrocephalus. PFS and OS were longer in patients with thalamic glioblastoma with a high resection degree (*P* < 0.05) and were not associated with hydrocephalus.

## Introduction

Thalamic tumors account for 5% of all brain tumors. The main pathological type is glioma, with lower proportions of lymphoma, cavernosum angioma, and inflammatory lesions (1–3). The pathological grade of thalamic glioma is low in pediatric patients and high in adults (4). The thalamus is located in the center of brain and connected with the hypothalamus, ventriculus tertius, lateral ventricles, basal ganglia, and aqueduct of the midbrain. As a result, surgery on the thalamus is technically difficult and associated with a high risk of postoperative complications, leading to unfavorable prognosis: high mortality and disability rate. Recent advances in neuroimaging and surgical techniques have made surgical resection of thalamic tumors feasible and have reduced the morbidity which associated with this approach (5–7). Surgical resection combined with adjunct therapies, such as radiotherapy and chemotherapy, improves overall survival (OS) (6, 7). However the incidence of postoperative complications such as limb dysfunction and hydrocephalus as well as the high cost remain challenging (6, 8, 9). Hydrocephalus is a common preoperative and postoperative complication during thalamic tumor surgery (10–12). At present, ventriculoperitoneal shunt surgery and third ventriculostomy are commonly used methods to avoid postoperative hydrocephalus (10–12). However, there are very few reports about the risk factors of hydrocephalus after thalamic surgery. So, we conducted the research of the risk factors of postoperative hydrocephalus to reduce the incidence of postoperative hydrocephalus and improve the prognosis and quality of life for thalamic tumor patients. Therefore, tumor resection needs to be extensive following standard treatments to reduce the incidence of postoperative hydrocephalus.

We performed a retrospective review of patients treated at our department for thalamic tumors in the last 6 years. We report our experience to address the following questions: the risk factors of early hydrocephalus after thalamic tumor resection and the effects of the surgical approach and surgical treatment strategy on early postoperative hydrocephalus. We also examined possible treatments to prolong OS and reduce postoperative hydrocephalus to improve mobility and motility.

## Materials and Methods

### Clinical Materials

We retrospectively reviewed 80 patients with thalamic tumors treated by tumor resection at Sanbo Brain Hospital, Capital Medical University, between January 1st 2015 and January 30th 2021. All cases in the research achieved surgical resection, and cases with stereotactic biopsy were not included in this research. The data included demographics, presenting symptoms, imaging characteristics, tumor extent, histology, treatment received, and follow-up (Table 1).

**Table 1:** Summary of preoperative clinical and radiological features in 80 patients.

**Characteristics**	***N***
**Ages**	
Median	33
Range	1–65
**Sex**	
Male	43
Female	37
**Initial case**	78
**Recurrent cases**	2
**Tumor site**	
Right	43
Left	37
**Preoperative maximum tumor diameter (mm)**	
Median	42
Range	12-78
**Preoperative Karnofsky Performance Status scores**	
Median	70
Range	20–90
**Symptoms and signs**	
Intracranial hypertension	38
Motor disturbance	15
Sensory dysfunction	4
Dizziness	11
Visual disfunction	4
Others	5

### Radiological Profiles

All patients underwent preoperative magnetic resonance imaging (MRI) (Table 2). The pre-operative MRI sequences included enhancement T1 weighted, T1 weighted, T2 weighted and flair imaging. Part of the patients achieved SWI and DTI imaging pre-operatively. SWI imaging examination can help to judge the deep veins around the tumor and protect these important structures intra-operatively. DTI imaging can help to judge the relationship between fiber bundle and tumor pre-operatively, and therefor reduce postoperative complications.

**Table 2:** Summary of preoperative imaging characteristics in 80 patients.

**Characteristics**	***N* (%)**
**Tumor location**	
Confined to the thalamus	21 (26.25%)
Invading the surrounding structures (brainstem, basal ganglia, contralateral thalamus)	59 (73.75%)
**Maximum tumor diameter (mm)**	
Median	42
Range	12–78
**Cystic change**	
Yes	23 (28.75%)
No	57 (71.25%)
**Enhancement in imaging**	
Homogeneous enhancement	11 (13.75%)
Partial enhancement	57 (72.25%)
Without enhancement	12 (15.00%)
**Peritumoral edema**	
Yes	21 (26.25%)
No	59 (73.75%)
**Preoperative hydrocephalus**	
Yes	64 (80.00%)
No	16 (20.00%)

### Treatment

Three microsurgery approaches were used according to the location of the tumor in the thalamus and surrounding structures.

If the main body of thalamic lesions is located in the thalamus and does not invade the lateral structure of the thalamus widely enough, we applied surgical resection through unilateral trans-cortical lateral ventricle approach. The patient was in supine position with head raised 30°. The frontal cortex site during the operation is located 2.5 cm in front of the coronal suture and 1 cm beside the midline. The diameter of the cortical ostomy is about 2.5 cm and the depth of the ostomy is about 3 cm. In most cases, the top of the thalamic tumor could be observed outside the choroid plexus of the lateral ventricle. If thalamic lesions invade the lateral structure of thalamus and grow posterolateral, we adopt surgical resection through parieto-occipital transventricular approach. The patient was placed in lateral position, and the incision center was located in the trigone of lateral ventricle. The body surface projection of the cortical ostomy was 7 cm beside the midline and 3 cm above the transverse sinus. The diameter of the cortical ostomy is 2.5–3 cm, and the depth is about 3 cm. With this ostomy the tumor could be sufficiently exposed.

**Figure 1:**
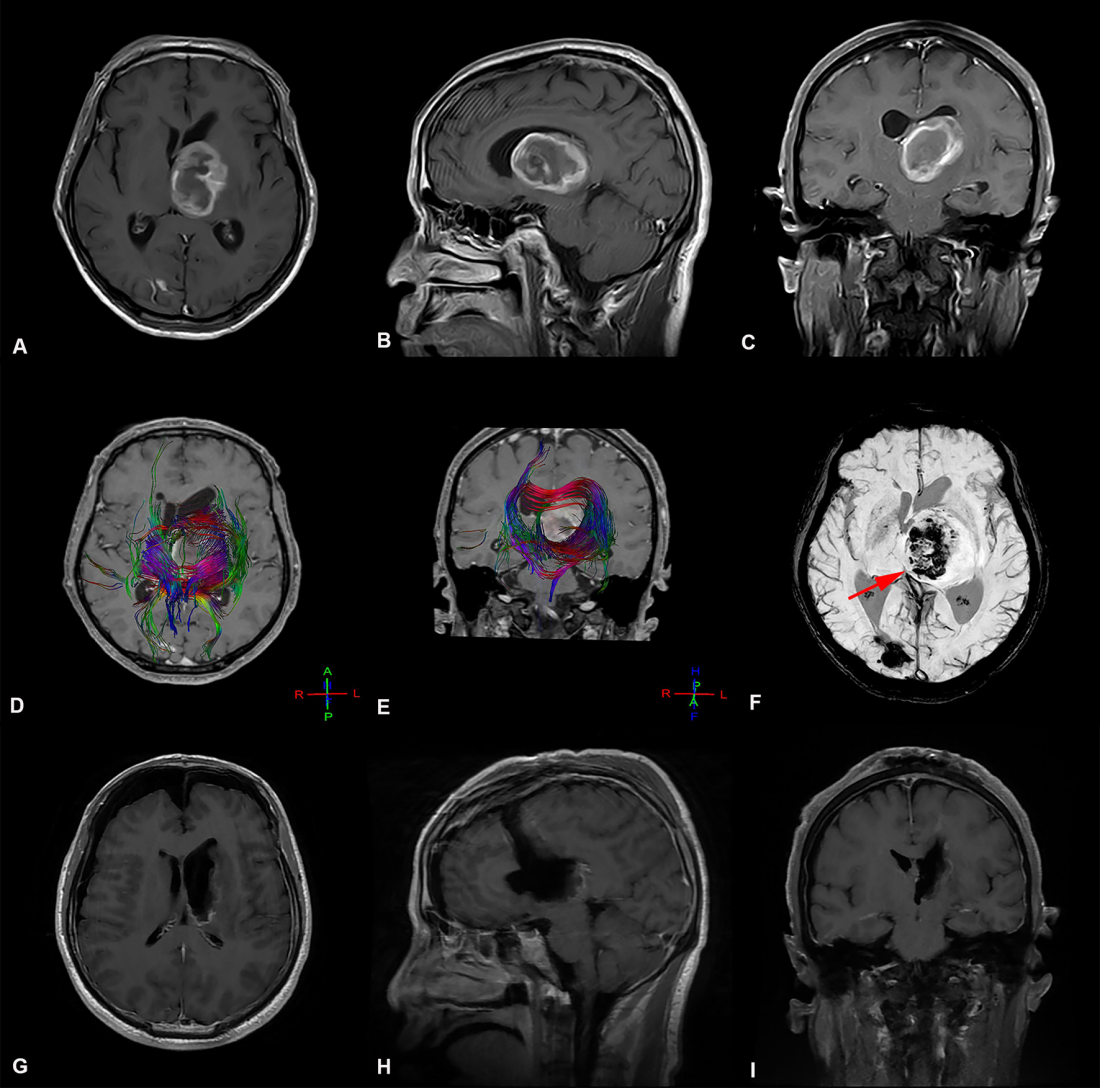
Preoperative and postoperative imaging of thalamic malignant tumor resection with trans-frontal lateral ventricle approach. **(A-C)** display: Axial, sagittal, and coronal enhanced MRI of malignant tumors of the left thalamus. The main body of the tumor is located in the thalamus, pushing the surrounding normal structure, supratentorial lateral ventricle expansion, and the third ventricle pushing to the right. **(D,E)** display: The bundle of fibers passing through the left thalamus is destroyed and pushed by the tumor. **(F)** The deep cerebral vein (great cerebral vein 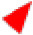) is located at the ventral and posterior side of the tumor and is pushed to the right by the tumor **(G-I)** display: MRI images obtained after thalamic tumor resection via the trans-frontal lateral ventricle approach. The images show that the volume of ventricles did not increase or expand after total tumor resection.

**Figure 2:**
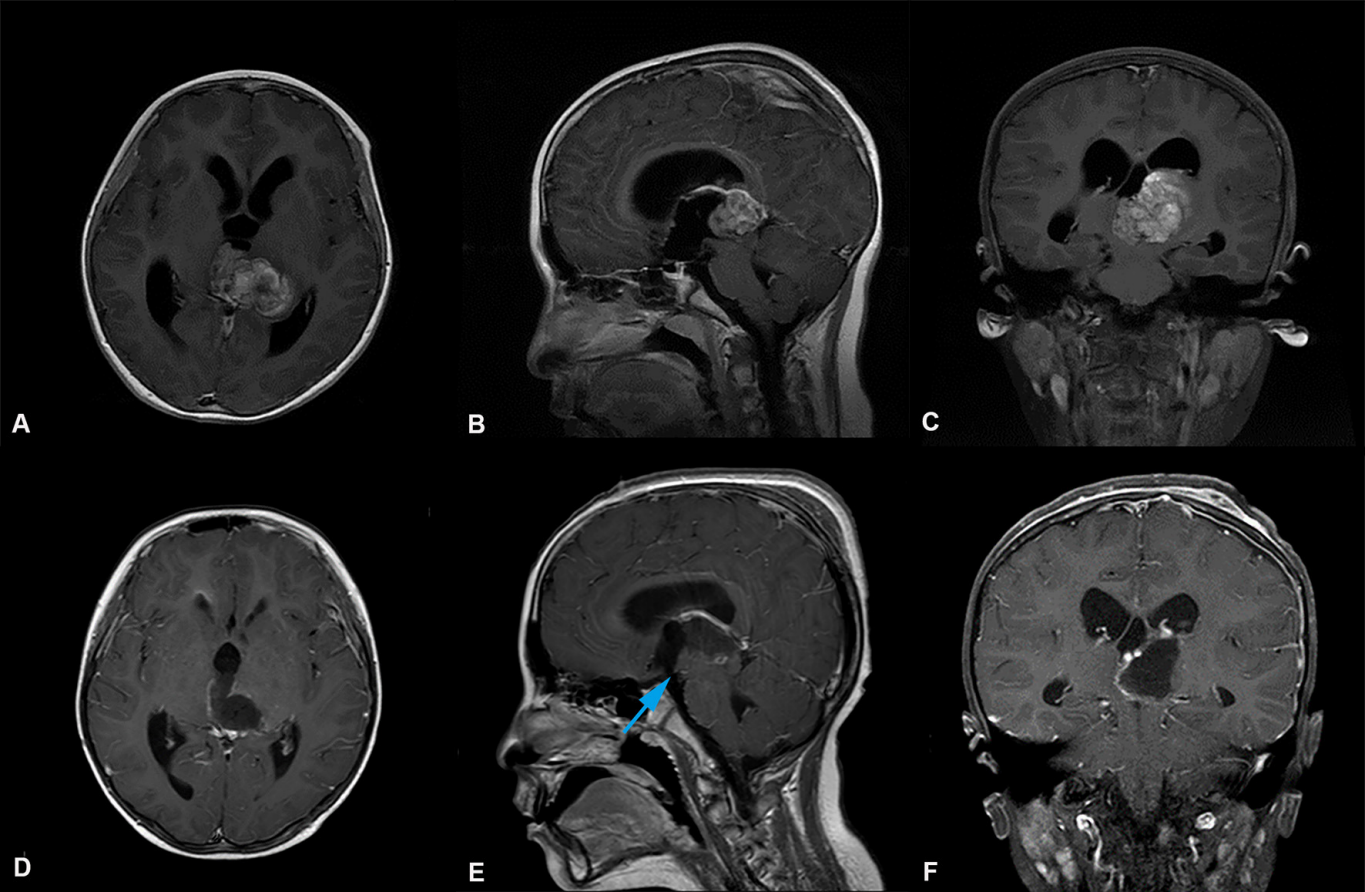
Preoperative and postoperative imaging of thalamic malignant tumor resection with left trans-frontal lateral ventricle approach + third ventriculostomy. **(A-C)** display: Axial, sagittal and coronal enhanced MRI of malignant tumors of the left thalamus. MRI shows that the main body of the tumor was located in the left thalamus, invading the third ventricle, the right thalamus, the supratentorial lateral ventricle, and the expansion of the third ventricle. **(D-F)** display: MRI of thalamic tumor resection with left trans-frontal lateral ventricle approach + third ventriculostomy. The images show that the tumor was completely removed, and the volume of the lateral ventricle did not increase and expand. The position indicated by the blue arrow (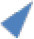) in e is the leak at the bottom of the third ventricle.

**Figure 3:**
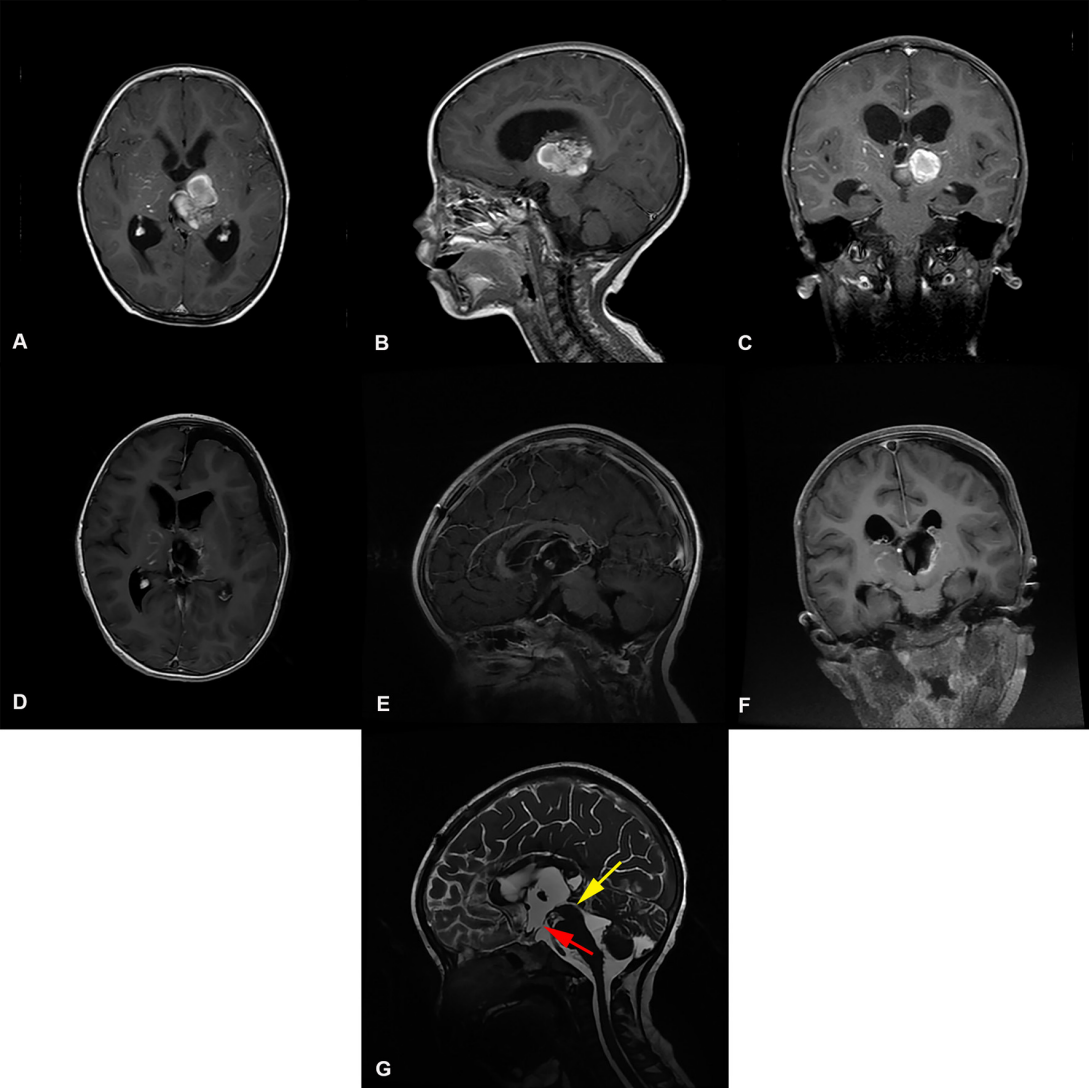
Preoperative and postoperative imaging of thalamic cavernous hemangioma resection with left trans-frontal lateral ventricle approach + third ventriculostomy. **(A-C)** display: Axial, sagittal and coronal enhanced MRI of benign tumors of the left thalamus. The MRI shows that the main body of the tumor was located in the left thalamus, invading the third ventricle, and the supratentorial lateral ventricle and third ventricle were dilated. **(D-F)** display: MRI images of thalamic tumor resection with left trans-frontal lateral ventricle approach + third ventriculostomy. The images show total resection of the tumor; the volume of the lateral ventricle after surgical resection of the thalamic lesion did not expand. **(G)** display: Three ventriculostomy was performed after resection of thalamic tumor The MRI T2 shows that the upper mouth of the midbrain aqueduct was thin, as indicated by the yellow arrow(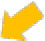). The position indicated by the red arrow (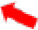) is the leak at the bottom of the third ventricle.

**Figure 4:**
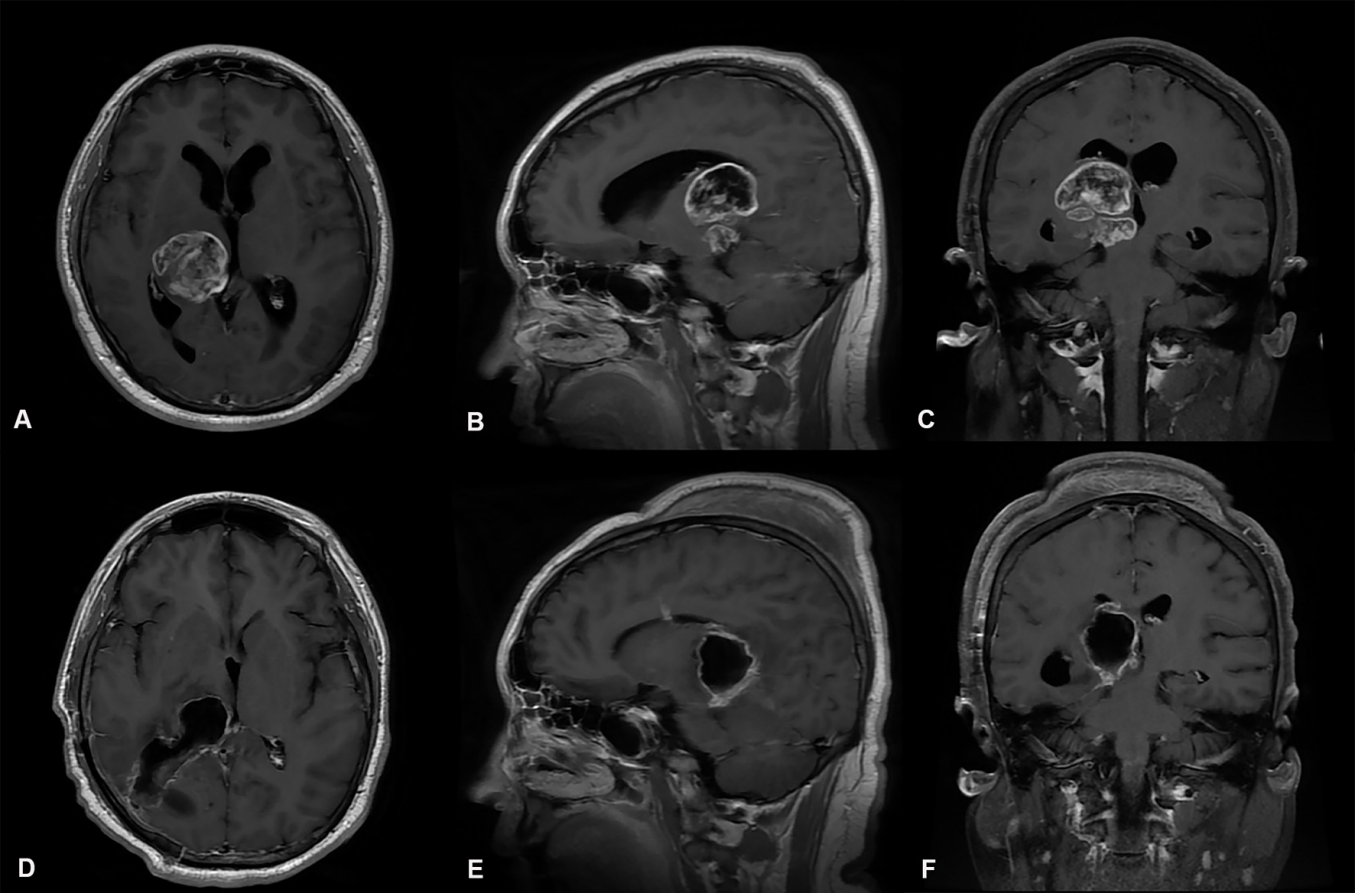
Preoperative and postoperative imaging of thalamic malignant tumor resection with through parieto-occipital transventricular approach **(A-C)** display: Axial, sagittal and coronal enhanced MRI of malignant tumors of the right thalamus. The MRI shows that the main body of the tumor was located in the right thalamus; the supratentorial lateral ventricle and third ventricle were dilated. **(D-F)** display: MRI of thalamic tumor resection with the right lateral ventricular triangle approach. The images show total resection of the tumor; the volume of the lateral ventricle after surgical resection of the thalamic lesion did not expand.

Tumor resection was performed via trans-frontal lateral ventricle approach for tumor resection in 27 cases (33.75 %) (Figure 1), trans-frontal lateral ventricle approach for tumor resection plus third ventriculostomy” in 14 cases (17.5%) (Figures 2, 3), and l through parieto-occipital transventricular approach tumor resection in 39 cases (48.75%) (Figure 4).

Patients with glioma diagnosed by postoperative pathology received chemotherapy and radiotherapy. The Stupp strategy was used in Class WHO IV (13).

### Follow Up

All follow-up MRI studies were performed at 1 month post-surgery, and then every 3–6 months. OS and the progression-free survival (PFS) data were calculated from the 1st day after operation. Disease progression was defined as symptom worsening. Tumor recurrence was evaluated by MRI or magnetic resonance spectroscopy (MRS). The post-operative MRI sequencess included T1 weighted, T2 weighted, enhanced T1 weighted and Flair imaging. Whether the ventricle was enlarged and whether there was hydrocephalus were observed. Some post-operatively patients underwent cerebrospinal fluid cine sequences. The sequence used for cerebrospinal fluid flow sequences examination is cerebrospinal fluid phase contrast sequence (CF-PCA, CSF-QF sequence). MRI examination was performed using the Netherlands Philips Archieva 1.5 T MR imaging system. RANO standard was used as the evaluation standard of treatment response of high-grade glioma. Post-operative MRI was performed to evaluate the surgical effect, whether the tumor recurred, whether the residual tumor volume increased, and whether new lesions appeared in other locations. MRS can help to distinguish tumor recurrence and radiation necrosis. Within 1 month and 3 months post-operatively, all patients achieved MRI to evaluate whether the tumor recurred and the effect of the followed treatment (such as chemotherapy and radiotherapy). Follow-up MRI was performed once every 6 months for benign tumors and every 3 months for malignant tumors. Follow-up time was to the end of life. The timing of follow-up MRI was of course adapted to possible modifications of clinical conditions. If hydrocephalus occurs in the early stage after surgery, ventriculoperitoneal shunt will be performed. We use Medtronic adjustable pressure shunt tube, and the initial value of pressure valve after shunt is 2.5 kpa, Subsequently, the pressure would be adjusted according to whether the patient had intracranial hypertension symptoms. There were no complications of infection and shunt tube dependent syndrome after shunt operation. If the postoperative imaging data showed that the ventricle was enlarged (it may be because there was hydrocephalus before the operation, resulting in the decrease of brain compliance, or incomplete obstruction after the operation. The ventricle size would not be improved after the operation), while the patient had no symptoms of high intracranial pressure, it would continue to be observed. For early hydrocephalus, the postoperative follow-up time continued until the case died.

### Statistical Analysis

SPSS 22.0 software (SPSS Inc., Chicago, IL, USA) was used for statistical analysis. Kaplan–Meier curves were used to calculate OS and PFS. multivariate analysis was performed by the log-rank test. Correlations were made using multivariate analysis according to the Kaplan–Meier method. The level of significance (p) was 0.05 for all tests.

## Results

### Extent of Surgical Resection

All the 80 cases underwent surgery. All patients survived during the perioperative period. We independently reviewed the preoperative and postoperative MRI data to determine the extent of tumor resection. The extent of resection was divided into gross total resection (90–100% removal; 44 cases), subtotal resection (80–90% removal; 21 cases), and partial resection (<80% removal; 15 cases) based on the intraoperative findings and postoperative MRIs (14). The volume of tumor was measured by “miplatform ZFP viewer” software. All radiological data, including pre-operative and post-operative data, were drawn directly from the peri-surgical reports and we invited a neuro-pathologist to check the validity. The neuro-radiologist was blinded to the results of pathological examination, surgical methods and clinical conditions. The neuro-radiologist have more than 10 years of image reading experience. Factors including tumor volume (unit: mm3), tumor location, scope, peritumoral edema, cystic change, calcification, peritumoral edema, deep vein, fiber bundle imaging Cine imaging of cerebrospinal fluid were evaluated. Besides whether there was hydrocephalus and distant metastasis were also evaluated.

### Histopathology

The pathological diagnoses of the 80 patients were as follows: 54 patients had glioblastoma (WHO IV), six had anaplastic astrocytoma (WHO III), seven were WHO II, one had diffuse large B lymphoma, one germinoma, one embryonal tumor of the central nervous system, and 10 had benign tumors. There were 10 cases of benign entities, i.e., five cases of cavernous hemangioma, one case of pilocytic astrocytoma, one case of brain abscess, one case of arachnoid cyst, one case of pilocytic hemangioma, and one case of epidermoid cyst.

### Treatment Outcomes

There were 35 cases (43.75%) of hydrocephalus at 1 month after operation. Among them, hydrocephalus occurred in seven cases after trans-frontal lateral ventricle approach for tumor resection (62.9%), in 17 cases after through parieto-occipital transventricular approach tumor resection (43.58%), and in one case after trans-frontal lateral ventricle approach for tumor resection + third ventriculostomy (7.1%) (Table 3).

**Table 3:** Relationship between different surgical approaches and postoperative hydrocephalus of thalamic tumors.

**Surgical approach**	**Number of cases**	**Number of postoperative hydrocephalus**
	***N* (%)**	***N* (%)**
Trans-frontal lateral ventricle approach plus third ventriculostomy	14 (17.50%)	1 (7.14%)
Trans-frontal lateral ventricle approach	27 (33.75%)	17 (62.96%)
Through parieto-occipital transventricular approach	39 (48.75%)	17 (43.59%)

The risk factors correlated with hydrocephalus were determined by multivariate analysis (*P* < 0.05) (Tables 4, 5). Hydrocephalus after unilateral thalamic space-occupying surgery is related to whether hydrocephalus is present before surgery, the degree of tumor resection, and whether third ventriculostomy is performed. Intraoperative third ventriculostomy can significantly reduce the symptoms of postoperative short-term hydrocephalus. The more extensive the surgical resection, the lower the risk of early postoperative hydrocephalus. If the patient has complications of hydrocephalus before the operation, the risk of hydrocephalus early after surgery is high.

**Table 4:** The risk factors correlated with hydrocephalus were determined by multivariate analysis (*P* < 0.05).

**Factor**	**OR**	**95%CI**	***P*-value**
Third ventriculostomy (Yes/No)	0.076	0.008–0.690	0.022
Degree of tumor resection	0.459	0.213–0.991	0.047
Tumor volume	1.008	0.995–1.020	0.246
Enhancement in imaging	0.830	0.284–2.419	0.732
Tumor crossing midline	0.324	0.050–2.110	0.238
Peritumoral edema	1.231	0.342–4.426	0.750
Benign and malignant tumor	2.232	0.272–18.295	0.454
Preoperative hydrocephalus	7.534	1.367–41.519	0.020

**Table 5:** Spearman multivariate analysis and correlation coefficient (*p* < 0.05).

**Factors**		**Correlation coefficient**	***P*-value**
Third ventriculostomy (Yes/No)	Degree of tumor resection	0.221	0.049
	Tumor volume	0.224	0.046
	Preoperative hydrocephalus	0.230	0.040
Degree of tumor resection	Enhancement in imaging	0.288	0.010
	Tumor crossing midline	−0.240	0.032
Tumor volume	Tumor crossing midline	0.489	<0.001
Enhancement in imaging	Peritumoral edema	0.226	0.044
Tumor crossing midline	Peritumoral edema	0.298	0.007
	Benign and malignant tumor	0.283	0.011
	Preoperative hydrocephalus	0.531	<0.001
Peritumoral edema	Benign and malignant tumor	0.225	0.044

The early occurrence of hydrocephalus after unilateral thalamic space-occupying surgery is not correlated with tumor volume, benign or malignant lesions, tumor image enhancement degree, peritumoral edema, invasion, tumor crossing midline, or pathological grade.

### Survival Analysis

**Figure 5:**
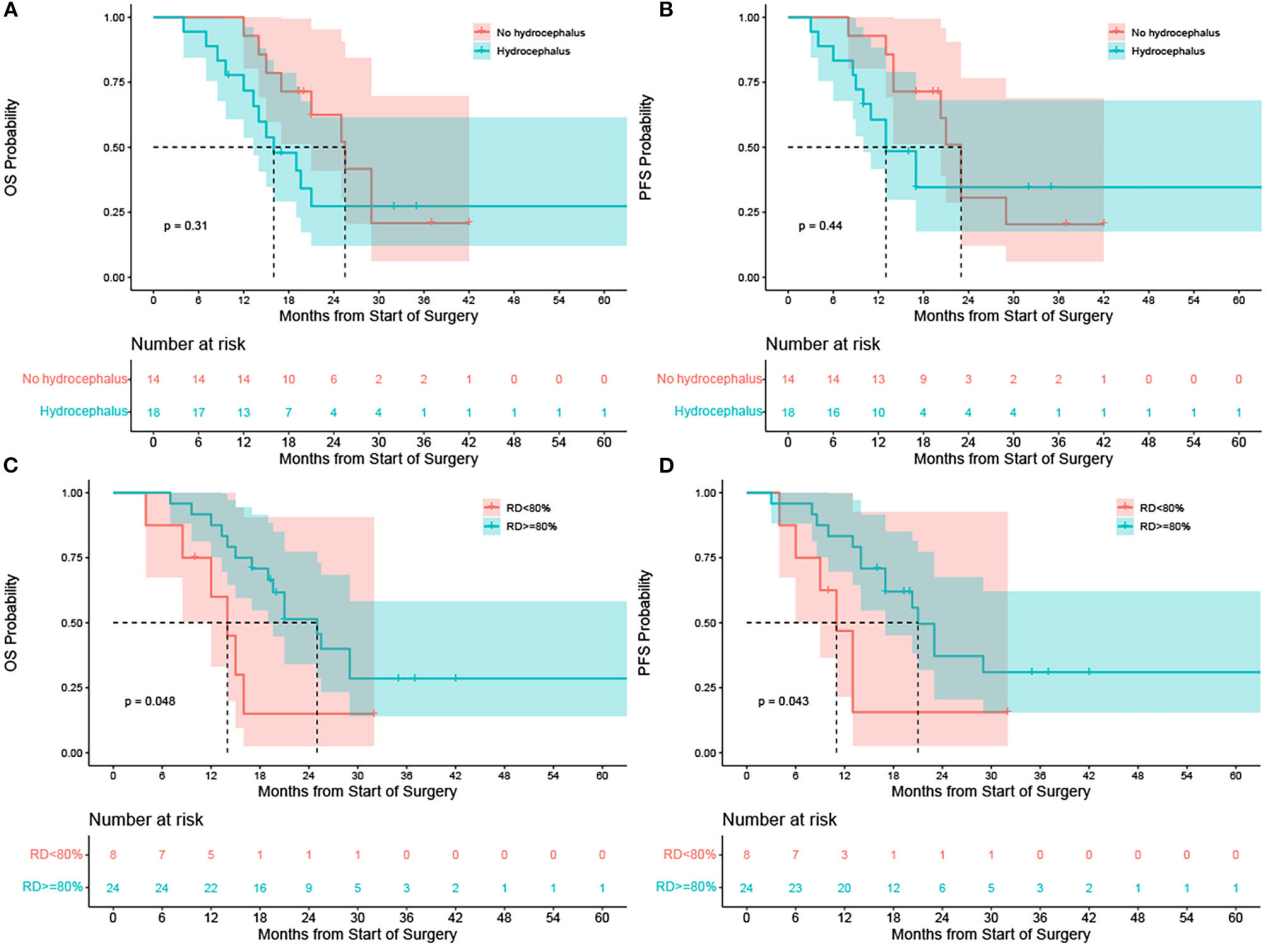
**(A)** Correlation of overall survival after tumor resection with early hydrocephalus in the glioblastoma group (green line: no hydrocephalus in the early postoperative period, blue line: hydrocephalus in the early postoperative period). The results showed that there was no correlation between early postoperative hydrocephalus and overall survival rate; *p* = 0.31. **(B)** Correlation between progression-free survival after tumor resection and early hydrocephalus in the glioblastoma group (green line: no hydrocephalus in the early postoperative period; blue line: hydrocephalus in the early postoperative period). The results show that there was no correlation between early postoperative hydrocephalus and progression-free survival rate; *p* = 0.44. **(C)** Correlation between tumor resection degree and overall survival rate in the glioblastoma group (green line: resection degree ≥80%; blue line: resection degree <80%). The results showed that full resection of lesions improved the overall survival rate (OS); *p* = 0.048. **(D)** Correlation between tumor resection degree and progression free survival rate in the glioblastoma group (green line: resection degree ≥80%; blue line: resection degree <80%). The results showed that full resection of lesions improved the total progression free survival (PFS); *p* = 0.043.

The survival time of thalamic glioblastoma is shorter than the other tumors with low malignancy. Glioblastoma accounts for the majority of cases in this research, Therefore, the prognosis of other tumors has not been systematically analyzed. In the glioblastoma group, hydrocephalus developing short-term after tumor resection (within 1 month) was not correlated with OS (Figure 5A) or PFS (Figure 5B) rate.

The glioblastoma group was divided into two subgroups, patients whose resection degree ≥80% and patients whose resection degree <80%. The results showed that resection degree ≥80% improved OS (Figure 5C) and PFS (Figure 5 D).

There is no dead case among all the benign cases, and only one case of cavernous hemangioma had hydrocephalus complications early after operation.

## Discussion

There are many surgical approaches to remove thalamic tumors, Several trans-midline approaches have been reported in the literatures for thalamus tumors (e.g., the interhemispheric transparaterminal gyrus approach described by Kumar and the transcallosal interforniceal approach described by Graziano) (14, 15).

There is really no large-scale clinical comparison between transcortical approach and transcallosal interfornix approach to show which approach is better, but we choose trans-cortical approaches mainly from the following points.

Compared to these reported approaches, the trans-cortical approach we chose contains the following advantages:

For unilateral thalamic lesions the tran-scortical approach could supply better surgical corridor, while the trans-midline approaches is suitable for lesions invading bilateral thalamus. Besides, as the fiber bundles of the corpus callosum are relatively dense, retraction of the corpus callosum would be more difficult and generate more injury.

Graziano proposed a novel approach: contractual antior inter hemispheric transitional gyrus approach (15). This approach is beneficial to the protection of corticospinal tracts. However, it also needs to cut a section of corpus callosum. In addition, this surgical channel will pass through many important structures, such as anterior communicating arteries, both A2 anterior cerebral arteries; Lastly, if the thalamic tumor grows to the top of the thalamus, the surgical field of vision may be poor when dealing with the superior part of the tumor through this approach.

Postoperative hydrocephalus leads to a decline in the patient's consciousness and increases the potential risk of hernia cerebri, which is life-threatening in severe cases. For postoperative hydrocephalus of the thalamus, a lateral ventricular puncture and external drainage may increase the risk of infection, and a lateral ventricular peritoneal shunt increases the medical burden. Therefore, it is important to analyze the risk factors related to hydrocephalus after thalamic tumor resection and reduce the risk of postoperative hydrocephalus.

**Figure 6:**
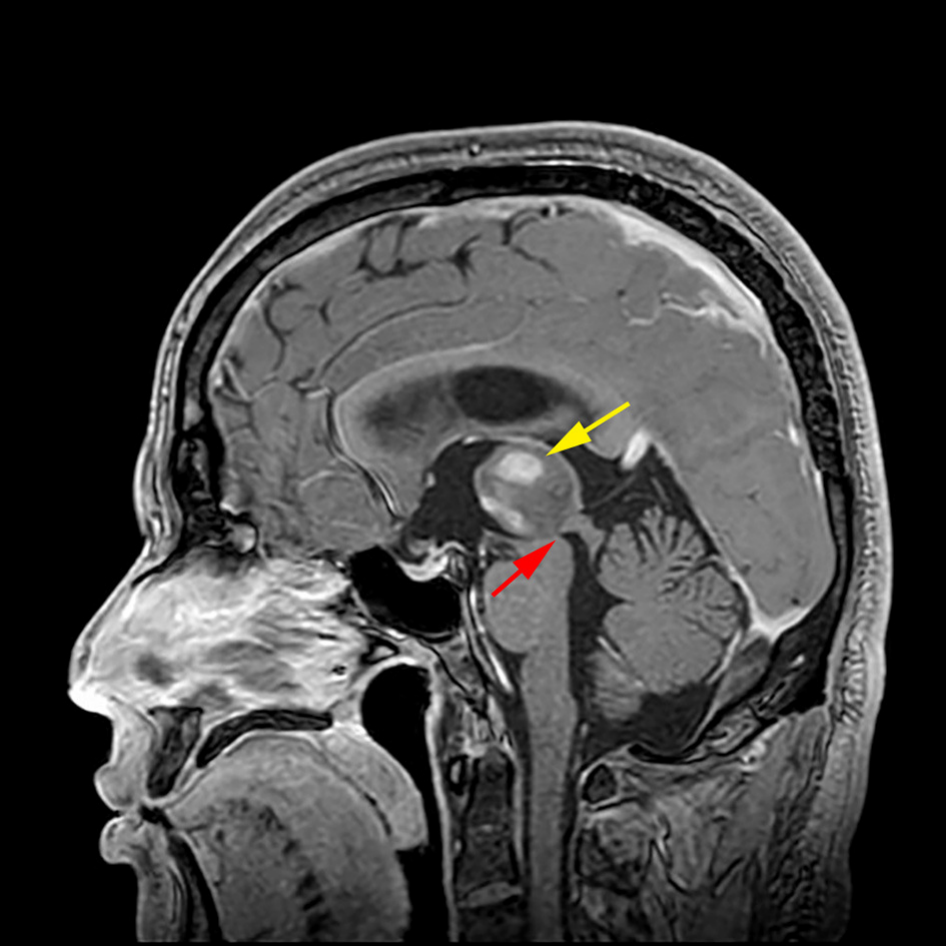
Mechanism of hydrocephalus caused by thalamic glioma. The right thalamic tumor (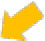 indicated) led to stenosis of the upper mouth of the midbrain aqueduct (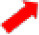 indicated), and the circulation of the hydrocephalus is blocked, resulting in obstructive hydrocephalus

To analyze the risk factors of postoperative hydrocephalus in thalamic lesions, we can clarify the problems according to our research results. First of all, we should clarify a question: what is the mechanism of hydrocephalus caused by thalamic space occupying lesions (Figure 6). Second, we need a clear view that the causes of hydrocephalus after thalamic space occupying surgery may be caused by the following reasons (1). The lesion was not completely resected, and the midbrain aqueduct was still blocked. (2) Scar formation around the upper end of the midbrain aqueduct after the operation. (3) The tumor recurred after the operation or continued to grow after partial resection.

Why is there a high risk of hydrocephalus after thalamic tumor resection with trans-frontal lateral ventricle approach in this group of cases? The reason is that the tumor body of the patient undergoing this operation is close to the midline. In this case, it is easy to cause infiltration and compression of the upper mouth of the midbrain aqueduct, and part of the tumor invades the third ventricle. Therefore, some of the tumor may remain in place after the lesion is resected, and a scar can form around the upper end of the midbrain aqueduct after the lesion is resected. In the triangular approach, the main body of the tumor is near the lateral thalamus and away from the midline structures, such as the midbrain aqueduct and third ventricle. Therefore, the probability of invading these structures is low, and the probability of hydrocephalus after lesion resection is low.

Why does third ventriculostomy after thalamic lesion resection reduce the risk of hydrocephalus after thalamic lesion resection? The third ventriculostomy adds an additional artificial circulation channel to the cerebrospinal fluid. Even in cases of midbrain aqueduct stenosis, hydrocephalus can be prevented if the ostomy at the bottom of the third ventricle is not closed.

Why is the number of thalamic lesions removed during surgery correlated with a lower risk of postoperative hydrocephalus? Full removal of the focus in the thalamus can eliminate the compression of the focus on the midbrain aqueduct and delay potential tumor growth that can continue to compress the midbrain aqueduct, which is helpful to reduce the risk of postoperative hydrocephalus. However, scar formation around the upper end of the midbrain aqueduct may cause stenosis of the midbrain aqueduct and lead to the occurrence of postoperative hydrocephalus.

Why is there a high risk of hydrocephalus after surgical resection of thalamic tumors with complications of hydrocephalus before operation? In cases of preoperative hydrocephalus, the high intracranial pressure causes dysfunction of the choroid plexus structure in absorbing and secreting cerebrospinal fluid, resulting in an imbalance between the secreting hydrocephalus and absorbing cerebrospinal fluid. The intracranial hypertension caused by the preoperative hydrocephalus decreases the compliance of brain tissue. Therefore, even if the obstructive factors causing hydrocephalus are relieved after the operation, the dysfunction of cerebrospinal fluid absorption and secretion and the decrease in brain tissue compliance may still lead to communicating hydrocephalus. The superposition of obstructive factors may also lead to hydrocephalus.

The special location of thalamic tumors can make resection difficult. Their treatment and the role of aggressive surgical resection have been a matter of debate for the last two decades (9, 16–20). Advances in neuroimaging and surgical techniques have made surgical resection of thalamic tumors feasible. Surgical resection in combination with adjunct therapies, such as radiotherapy and chemotherapy, based on the histological characteristics of the tumor has been suggested by many publications (7–9, 13, 16–26). However, postoperative complications such as limb dysfunction and hydrocephalus remain challenging (6, 8, 9, 27). Solving these problems is important to improve mobility and motility.

Postoperative hydrocephalus may lead to coma and cerebral hernia, which are life-threatening. Treatment of hydrocephalus may further cause infection and is associated with a high cost. Therefore, decreasing the incidence of postoperative hydrocephalus is important.

Postoperative hydrocephalus may be caused by incomplete resection, scar contracture, anastomotic stenosis, and the tumor type. Patients with a trans-frontal lateral ventricle approach are prone to postoperative hydrocephalus. The tumor in this group is located near the midline, compressing the midbrain aqueduct and third ventricle. Scar contracture and anastomotic stenosis often occur after surgery. Tumors from patients who undergo through parieto-occipital transventricular approach are located laterally from the thalamus, away from the midbrain aqueduct and third ventricle. This location is associated with a low incidence of hydrocephalus. The third ventriculostomy bypasses fluid despite compression of the midbrain aqueduct, which decreases the risk of hydrocephalus. Gross total resection relieves the compression of the midbrain aqueduct to reduce the incidence of hydrocephalus. Preoperative hydrocephalus is associated with high intracranial pressure, which affects the ability of the choroid plexus to absorb cerebrospinal fluid, thus increasing the risk of postoperative hydrocephalus despite tumor resection.

## Conclusion

Surgical treatment of thalamic tumors is an effective therapeutic method. The incidence of postoperative hydrocephalus is not associated with factors including tumor size, degree of tumor enhancement, peritumoral edema, tumor invasion, midline crossing, and pathological grade. The incidence of postoperative hydrocephalus is higher in patients with preoperative hydrocephalus and low resection degree, meanwhile patients with third ventriculostomy got lower postoperative hydrocephalus risk. Besides, the risk of early postoperative hydrocephalus in thalamic tumors is high. Intraoperative triple ventriculostomy can reduce the incidence of early postoperative hydrocephalus. PFS and OS are longer in thalamic glioblastoma cases with a high resection degree.

## Data Availability Statement

The original contributions presented in the study are included in the article/supplementary material, further inquiries can be directed to the corresponding author/s.

## Ethics Statement

The studies involving human participants were reviewed and approved by Ethics Committee of Sanbo Brain Hospital. Written informed consent for publication was obtained from all participants. Written informed consent to participate in this study was provided by the participants' legal guardian/next of kin. Written informed consent was obtained from the individual(s), and minor(s)' legal guardian/next of kin, for the publication of any potentially identifiable images or data included in this article.

## Author Contributions

XZ designed the study. LZ and CW collected data and revised the manuscript for important intellectual content. LZ analyzed the data and wrote the article. All authors have read and approved the final manuscript.

## Conflict of Interest

The authors declare that the research was conducted in the absence of any commercial or financial relationships that could be construed as a potential conflict of interest.

## Publisher's Note

All claims expressed in this article are solely those of the authors and do not necessarily represent those of their affiliated organizations, or those of the publisher, the editors and the reviewers. Any product that may be evaluated in this article, or claim that may be made by its manufacturer, is not guaranteed or endorsed by the publisher.
